# The Relationship Between Precuneus Thickness and Psychopathology in Adolescent Females With Anorexia Nervosa

**DOI:** 10.1002/eat.24591

**Published:** 2025-11-03

**Authors:** Irina Jarvers, Raphael Degmayr, Alexandra Otto, Ricarda Jacob, Wilhelm Malloni, Stephanie Kandsperger, Daniel Schleicher, Angelika Ecker, Isabel Wiesinger, Christina Wendl, Mark Greenlee, Romuald Brunner

**Affiliations:** ^1^ Department of Child and Adolescent Psychiatry and Psychotherapy University of Regensburg Regensburg Germany; ^2^ Department of Cognitive Neuroscience University of Regensburg Regensburg Germany; ^3^ Institute of Diagnostic Radiology, Interdisciplinary Ultrasound Department University Hospital Regensburg Regensburg Germany; ^4^ Department of Experimental Psychology University of Regensburg Regensburg Germany

**Keywords:** adolescents, alexithymia, anorexia nervosa, cortical surface area, cortical thickness, cortical volume, precuneus, psychopathology

## Abstract

**Objective:**

Anorexia nervosa (AN) is a severe psychiatric disorder with structural brain alterations; however, the roles of cortical surface area (CSA) and cortical thickness (CTh) during adolescence remain unclear. Building on frequent reports of gray matter reductions in the cingulate cortex and precuneus, this study assesses CSA, CTh, and cortical volume in these regions, alongside exploratory whole‐brain analyses and their associations with psychological dimensions.

**Method:**

We included 26 adolescent females aged 12–19 years with acute AN and 24 age‐matched healthy controls. Participants underwent high‐resolution structural MRI and completed psychological assessments: Eating Disorder Inventory‐2 (EDI‐II), Beck Anxiety Inventory (BAI), Beck Depression Inventory‐II (BDI‐II), Perth Alexithymia Questionnaire (PAQ), and Body Perception Questionnaire. MRI data were processed using BIDScoin, fMRIPrep, and FreeSurfer. Analyses included permutation‐based non‐parametric testing and multiple regression to investigate associations between brain metrics and psychological scores.

**Results:**

In primary analyses, individuals with AN exhibited a significant reduction in precuneus CTh only. Precuneus CTh correlated negatively with EDI‐II, BAI, and BDI‐II scores, and positively with BMI‐SDS. Regression analysis identified anxiety (BAI), specific EDI‐II subscales, supra‐diaphragmatic autonomic reactivity and difficulties describing negative feelings (PAQ) as predictors of precuneus CTh. Exploratory analyses revealed thickness differences in parietal and frontal regions, consistent with prior large‐scale studies, with anxiety and depression scores correlating with several of these regions.

**Discussion:**

Reduced precuneus CTh and its association with psychological factors highlight its role in AN's neurobiological mechanisms. Exploratory findings implicate parietal and frontal alterations, suggesting broader disruptions in body perception and behavioral control.

**Summary**

- This study examines cortical thickness (CTh), surface area, and volume in adolescents with anorexia nervosa (AN) using permutation‐based non‐parametric combination.
- Adolescents with AN show reduced precuneus CTh compared to healthy controls.
- Precuneus thickness is linked to psychological distress, BMI‐SDS, and emotional processing difficulties.
- Findings suggest malnutrition‐related brain changes may contribute to psychological symptoms in AN, highlighting the need for nutritional rehabilitation.

## 1 Introduction

Anorexia nervosa (AN) is characterized by persistent caloric restriction, an intense fear of weight gain, and a distorted body image (American Psychiatric Publication 2013), resulting in a severe psychiatric disorder predominantly affecting young women with numerous physical complications and a high mortality rate (Smink et al. 2012; Westmoreland et al. 2016; Arcelus et al. 2011).

Recent advances in brain imaging have shed light on the neurobiological underpinnings of AN. Brain magnetic resonance imaging (MRI) studies revealed that individuals with AN exhibit significant structural brain changes compared to healthy controls (Seitz et al. 2014; Yu et al. 2024). These changes are primarily characterized by decreased grey matter volume (GMV) in several brain regions critical for emotional regulation, cognitive control, and sensory processing (Walton et al. 2022). Zhang and colleagues performed a meta‐analysis of 21 MRI studies in 2018—including 389 individuals with AN and 410 healthy controls, both adolescents and adults—to characterize GMV alterations in individuals suffering from AN (Zhang et al. 2018). In this pooled comparison, significant reductions in GMV have been observed in the bilateral midcingulate cortex (MCC), posterior cingulate cortex (PCC), supplementary motor area, precuneus, cerebellum, left amygdala, and left anterior cingulate cortex (ACC) (Zhang et al. 2018; Su et al. 2021). The ACC, MCC, and PCC are all part of the cingulate cortex, which lies above the corpus callosum. Moreover, the cingulate cortex is part of the paralimbic system, integrating external stimuli with internal emotional and psychological states (Clark et al. 2018).

Building on these widespread GMV reductions, Zhang and colleagues hypothesized specific relationships between cortical grey matter volume decrease and its dysfunction in AN (Zhang et al. 2018). The ACC is implicated in reward networks and affective processing (Holliday et al. 2005; Bush et al. 2000) and may contribute to deficits related to set‐shifting, a neuropsychological trait observed in AN (Holliday et al. 2005). Functional MRI studies conducted by Zastrow and colleagues in adult AN patients have indicated reduced activity in the ACC (Zastrow et al. 2009). More posterior in the brain, the MCC also shows volumetric decreases in adolescents and adults with AN (Joos et al. 2010; Gaudio et al. 2011). The MCC, particularly its anterior subregion with heavy amygdala input, mediates emotional salience appraisal and fear‐avoidant behaviors (Vogt et al. 1992). This aligns with the emotional disturbances observed in AN (Zhang et al. 2018), such as anxiety, depression, and alexithymia—defined as difficulty identifying and describing one's own emotions along with an externally oriented thinking style that neglects internal emotional states (Preece et al. 2023). Further back, the PCC and the overlying precuneus are integral components of the default mode network (DMN), implicated in self‐reflection and self‐monitoring (Buckner et al. 2008; Lou et al. 2004). Reduced DMN activity, as seen in adult AN patients (McFadden et al. 2014) and AN patients in their young adulthood (Sachdev et al. 2008), potentially underpins the rigid self‐reflective loops and starvation denial that are characteristic of the disorder (Zhang et al. 2018). Finally, volumetric reductions in the amygdala itself (Giordano et al. 2001) may compound deficits in emotional regulation and threat processing (Phelps and LeDoux 2005; Davis and Whalen 2001). Together, these regional and network‐level disturbances may form a coherent neurobiological framework for understanding the core cognitive and emotional symptoms of AN.

Despite numerous studies demonstrating structural brain alterations in AN, most research has focused on adults (Seitz et al. 2014; Zhang et al. 2018; Zastrow et al. 2009; Joos et al. 2010; McFadden et al. 2014; Giordano et al. 2001). While adult studies have provided valuable insights into the neurobiological underpinnings of AN, adolescence is a critical period for brain development, marked by ongoing changes in cortical organization, cognitive function, and emotional regulation (Konrad et al. 2013). Given that AN often emerges during adolescence (Uhlhaas et al. 2023), understanding structural brain changes at this stage in neural development is essential for early intervention and treatment protocols. However, studies specifically examining adolescents with AN remain limited, with many focusing solely on overall GMV (Gaudio et al. 2011).

Cortical volume in the brain is commonly evaluated using two measures: cortical surface area (CSA) and cortical thickness (CTh). These measures are influenced by distinct genetic and developmental factors and have varying impacts on cognitive development and disorders (Winkler et al. 2010). However, combining CSA and CTh might oversimplify their individual contributions, potentially masking specific structural changes (Panizzon et al. 2009). Despite numerous studies examining overall cerebral volume in AN, there remains a need for focused research on the distinct roles of CSA and CTh in this disorder. Especially since the conducted studies on CSA and CTh in AN have yielded conflicting results (Myrvang et al. 2020). Additionally, brain volume is traditionally measured voxel‐by‐voxel or by multiplying CSA by CTh at each vertex. However, these methods can be sensitive to artifacts (Ashburner and Friston 2000) or may overestimate or underestimate volume (Winkler et al. 2018). A recent method, the permutation‐based nonparametric combination (NPC), integrates CTh and CSA metrics more effectively than the traditional volume analysis (Winkler et al. 2016). It independently tests CTh and CSA and synthesizes their statistics across permutations to generate a joint distribution. From this distribution, the final *p*‐value is derived, which is a robust statistic that requires fewer assumptions and acknowledges shared environmental influences (Winkler et al. 2016). Indeed, Winkler and colleagues showed that NPC outperforms the conventional volume metric (area × thickness) (Winkler et al. 2018). As described above, NPC examined surface area and CTh separately and then combines them with equal weight. This joint test increases statistical power and reveals group differences that single‐metric or simple volume analyses can miss (Winkler et al. 2016; Fisher 1992; Pesarin and Salmaso 2010), especially when area and thickness change at different rates or in opposite directions, as seen in brain development during adolescence (Hogstrom et al. 2013; Storsve et al. 2014). Therefore, NPC provides a more precise and sensitive way to quantify cortical changes in adolescent individuals with AN than traditional volumetric methods.

The pronounced GMV reductions identified by Zhang and colleagues and their proposed role in AN's core cognitive and emotional symptoms (Zhang et al. 2018) underscore the need to test whether these morphometric alterations relate to the psychopathology of AN using multiple targeted questionnaire‐based assessments in various psychological domains. Furthermore, given that adolescence represents a critical window for neurodevelopment and is a particularly sensitive period for the onset of AN (Uhlhaas et al. 2023), it is essential to explore these neural changes in the context of this developmental stage. Especially considering the complex interplay between cognitive, emotional, and sensory processes in AN, a multidisciplinary approach is needed. Therefore, we combine structural neuroimaging with psychological assessment data—including questionnaires on eating disorder symptoms, depression, anxiety, body perception, and alexithymia—to comprehensively understand and address the neural and psychological dimensions of AN.

In addition to our primary focus on the cingulate cortex and precuneus, we also evaluated global measures of brain structure (overall gray and white matter volumes) as a secondary objective. Furthermore, to capture potential alterations beyond our a priori regions of interest, we conducted an exploratory whole‐brain analysis.

We hypothesized that adolescent patients with AN would exhibit reduced GMV, CTh, and CSA in areas of the cingulate cortex and precuneus. Furthermore, we expected these structural alterations to be significantly associated with scores on the above‐mentioned psychological assessments.

## 2 Materials and Methods

### 2.1 Participants

The study included female patients aged 12–19 years diagnosed with AN (ICD‐10: F50.00, F50.01, F50.1) and a BMI below the 10th age percentile (*n* = 45). BMI and questionnaires were collected at T1, a median of 10.5 days before the MRI scan (T2). A total of *n* = 13 participants had to be excluded due to dental braces, *n* = 5 due to scan distortions shortly before and after a scanner update, and an additional *n* = 1 due to being above the 10th percentile during the measurement. Recruitment took place between December 14, 2021, and May 9, 2025. Exclusion criteria were carefully defined to ensure a homogeneous study sample. Individuals with neurological disorders, developmental conditions such as autism, substance dependence, intellectual disabilities, or contraindications for MRI were excluded. However, patients with frequent AN‐comorbidities, such as depression, anxiety, and obsessive–compulsive disorder were included. Patients were recruited at admission from the outpatient clinics, daycare setting, and inpatient wards of the Clinic and Polyclinic for Child and Adolescent Psychiatry, Psychosomatics, and Psychotherapy of the University of Regensburg, at the District Hospital Regensburg. For the control group, healthy female participants, matched by age, were recruited from the general population (*n* = 33). A total of *n* = 3 participants had to be excluded due to dental braces, *n* = 5 due to scan distortions shortly before and after a scanner update, and an additional *n* = 1 was excluded due to showing clinically significant symptomatology during the diagnostic interview. After these exclusions, complete data were available for all remaining participants in the patient group (*n* = 26) and control group (*n* = 24). Recruitment was conducted via flyers, posters, and newsletters distributed to hospital staff and youth centers. A structured clinical interview, the Mini International Neuropsychiatric Interview for Children and Adolescents (M.I.N.I.‐KID 6.0, (Sheehan et al. 1998)), ensured that control participants had no history of psychiatric disorders. All reported comorbidities of the patient group were based on expert clinical assessments in multidisciplinary teams conducted in the associated clinic. Recruitment for both groups began after obtaining ethical approval from the Ethics Committee of the University of Regensburg on July 23, 2021 (reference number: 21‐2438‐101). The study is preregistered in the German Clinical Trials Register (DRKS00025963). Data from this study have been previously published by Jarvers et al. (2023), examining the impact of the COVID‐19 pandemic on AN symptomatology. The present analyses answer a different question by focusing on the CTh of the cingulate cortex and precuneus and their associations with psychological dimensions of AN.

### 2.2 Measures

#### 2.2.1 Assessment of Eating Disorder Symptomatology

The Eating Disorder Inventory‐2 (EDI‐II) is a self‐report questionnaire designed to evaluate behavioral and psychological symptoms associated with eating disorders (Kappel et al. 2012). It is applicable to adolescent girls diagnosed with AN as well as to control groups. The EDI‐II consists of 91 items, each rated on a 6‐point Likert scale, and these items are categorized into 11 scales: drive for thinness, bulimia, body dissatisfaction, ineffectiveness, perfectionism, interpersonal distrust, interoceptive awareness, maturity fears, asceticism, impulse regulation, and social insecurity. The EDI‐II has demonstrated high internal consistency, good test–retest reliability and good validity (Kappel et al. 2012; Garner et al. 1983; Thiel and Paul 2006). Also, in adolescence, the measure largely follows the suggested six‐factor structure (Salbach‐Andrae et al. 2010). In the present sample the EDI‐II had an excellent internal consistency (*α* = 0.98) for the total score and ranged between 0.78 and 0.94 for the subscales with the exception of the Bulimia subscale (*α* = 0.57).

#### 2.2.2 Assessment of Anxiety Symptomatology

Beck's Anxiety Inventory (BAI) (Beck et al. 1988; Margraf and Ehlers 2007) was employed to assess anxiety symptoms. This self‐report questionnaire measures cognitive, emotional, and behavioral symptoms of anxiety. It comprises 21 items, each rated on a scale from 0 (not at all) to 3 (severely). The BAI has shown good internal consistency and test–retest reliability (Beck et al. 1988; Margraf and Ehlers 2007). In the present sample, the BAI had a Cronbach's α of 0.92.

#### 2.2.3 Assessment of Depression Symptomatology

Beck's Depression Inventory‐2 (BDI‐II) (Beck et al. 1961; Hautzinger et al. 2009) was used to evaluate depressive symptoms. Like the BAI, this self‐report questionnaire assesses cognitive, emotional, and behavioral symptoms, but focuses on depression. It also consists of 21 items rated on the same 4‐point scale, ranging from 0 (not at all) to 3 (severely). The BDI‐II is frequently used with adolescents aged 13 and older and has demonstrated excellent internal consistency and good test–retest reliability (Keller et al. 2022). In the present sample, the BDI had a Cronbach's α of 0.97.

#### 2.2.4 Assessment of Alexithymia

Alexithymia was assessed using the German version of the Perth Alexithymia Questionnaire (PAQ) for children and adolescents (Jarvers et al. 2021; Preece et al. 2018). The PAQ includes 24 items divided into three primary subscales: difficulties identifying feelings (DIF), difficulties describing feelings (DDF), difficulties appraising feelings (DAF), and an externally oriented thinking style (EOT). Additionally, compound scores can be generated by combining DIF, DDF, and DAF, along with additional scores that distinguish between difficulties with positive (PDIF, PDDF and PDAF) or negative emotions (NDIF, NDDF, and NDAF). Each item is rated on a 7‐point Likert scale, ranging from 1 (strongly disagree) to 7 (strongly agree). In the present sample, the PAQ total score had a Cronbach's α of 0.96 and ranged between 0.89 and 0.98 for all subscales.

#### 2.2.5 Assessment of Body Perception

The BPQ‐SF (Body Perception Questionnaire‐Short Form) (Cabrera et al. 2018) comprises 46 items evaluated on a 5‐point Likert scale with three subscales. The Body Awareness subscale (BPQ‐A), consisting of 26 items, measures how often individuals are conscious of bodily sensations. The other two subscales assess autonomic nervous system reactivity. The supradiaphragmatic reactivity subscale (BPQ‐supra), with 15 items, focuses on the responses of organs above the diaphragm. The subdiaphragmatic reactivity subscale (BPQ‐sub), comprising 6 items, targets the gastrointestinal responses. One item, related to the feeling of nausea, is included in both reactivity subscales. These subscales help identify challenges in bodily function coordination, stress symptoms, and autonomic dysregulation. The BPQ‐SF displayed good psychometric properties in previous studies (Cabrera et al. 2018; Brand et al. 2023). In the present sample, the BPQ had a Cronbach's α of 0.97 for BPQ‐A, 0.90 for BPQ‐supra, and 0.87 for BPQ‐sub.

### 2.3 Study Procedure

As part of an ongoing longitudinal study, participants took part in two separate appointments before weight restitution: a psychometric assessment (T1) and an MRI examination (T2).

The psychometric assessment included a standardized psychiatric and neuropsychological evaluation through a comprehensive set of tests. The study began with a semi‐structured baseline interview to collect study‐relevant data such as age, date of birth, weight, height, type of school, treatment setting, duration of illness, and previous medication. In addition, the M.I.N.I.‐KID (Sheehan et al. 1998) was conducted to assess mental health problems in the control group and provide an additional control instance for patients. Several questionnaires were administered, including the EDI‐II to evaluate eating disorder psychopathology, the BDI‐II for depressive symptoms, and the BAI to screen for anxiety symptoms. Additionally, the BPQ was used to measure individuals' awareness of bodily sensations, while the PAQ assessed alexithymia.

Following the psychometric assessment (T1), participants attended a separate MRI appointment (T2) a median of 10.5 days later. Anatomical data collection included a T1‐weighted scan and whole‐brain diffusion‐weighted imaging performed with a 64‐channel head coil on a 3 Tesla Siemens scanner. For this study, only the anatomical data obtained from the T1‐weighted scan were used for further analysis.

### 2.4 Structural MRI Image Pipeline

The T1‐weighted images were acquired using the MP‐RAGE (3D “magnetization prepared rapid gradient echo”) sequence resulting in 208 slices with a resolution of 0.8 × 0.8 × 0.8 mm^3^. Moreover, a FOV of 256 × 256 mm^2^, TR of 2400 ms, TE of 2.18 ms, TI of 1200 ms, and a flip angle of 8 degrees were used for a total scan duration of 6 min and 38 s. This dataset was first standardized to the Brain Imaging Data Structure (BIDS) format using BIDScoin, utilizing *bidsmapper*, *bidseditor*, and *bidscoiner* (BIDScoin 2024). Following this, the data underwent preprocessing with default settings of *fMRIPrep* in its docker version. *fMRIPrep* is an open‐source software designed to enhance the preprocessing of MRI images by integrating the most effective tools from *SPM* (Statistical Parametric Mapping), *FSL* (FMRIB Software Library), *AFNI* (Analysis of Functional Neuroimages), *ANT* (Advanced Normalization Tool), and *FreeSurfer* (Esteban et al. 2018). For the present study, preprocessing of T1‐weighted scans included N4 bias field correction and ANTs‐based skull stripping (OASIS30), followed by tissue segmentation into CSF, GM, and WM using FSL FAST. Cortical surfaces were reconstructed with FreeSurfer, with brain masks reconciled using Mindboggle and pial surfaces refined with FLAIR. The resulting images were nonlinearly normalized to both MNIPediatricAsym (cohort‐6) and MNI152NLin2009cAsym templates from TemplateFlow using ANTs. Lastly, *FreeSurfer*'s commands *asegstats2table* and *aparcstats2table* were used to create text (.txt) files containing volumetric, thickness, and surface area measurements of various brain regions (aparcstats2table 2024; asegstats2table 2024). For volume analyses, left and right hemisphere values were summed; for thickness and surface area, mean values across hemispheres were calculated. This ROI‐based morphometric approach, conducted in native space, aligns with previous clinical and methodological studies emphasizing subject‐specific anatomical accuracy and avoiding distortions from spatial normalization (Allen et al. 2008; Hutchison et al. 2014; Aribisala et al. 2011).

### 2.5 Statistical Analysis

Data analysis was conducted using *IBM SPSS Statistics* (Version 29). Normality was assessed using the Shapiro–Wilk test, and homogeneity of variances was evaluated with Levene's test. Depending on these outcomes, group comparisons were performed using either a two‐tailed *t*‐test, Welch's *t*‐test, or Mann–Whitney *U* test. Statistical significance was defined as *p* < 0.05, and multiple comparisons were controlled with the False Discovery Rate (FDR) (Benjamini and Hochberg 1995). Effect sizes are reported as Cohen's *d* for *t*‐tests (interpreted as small = 0.2, medium = 0.5, large = 0.8), and as *r* = Z/√N (small = 0.1, medium = 0.3, large = 0.5) (Cohen 1988).

First, we compared clinical and psychological measures between individuals with AN and controls. Second, for an overall survey of brain volume alterations, global brain metrics (total gray matter volume, total white matter volume, and total intracranial volume) and regional brain volumes such as the cortex, cerebellum, amygdala, and hippocampus were compared between the AN group and the control group utilizing a non‐parametric permutation test with 5000 iterations and the Mann–Whitney *U* test; *p*‐values were then FDR‐adjusted. Given our primary focus on the cingulate cortex and precuneus, analyses next concentrated on these regions. Third, a non‐parametric permutation test with 5000 iterations was used to compare the surface area and thickness of regions within the cingulate cortex and the precuneus between groups. Fourth, thickness and area *p*‐values were combined per subdivision of the cingulate cortex and precuneus using Fisher's method, and empirical *p*‐values were derived by comparing observed statistics against the permutation distribution. Final *p*‐values were FDR‐corrected across regions (Tukey 1991). To evaluate statistical power, Monte Carlo simulations with 600 simulated datasets and 1200 random permutations per region were performed, following recommendations from previous work (Bonnini et al. 2024; Marozzi 2004).

Fifth, for brain metrics showing significant group differences—in our sample, precuneus thickness—we calculated Kendall's tau correlations with total scores of all psychological assessments across the full sample. We additionally tested correlations between precuneus thickness and BMI Standard Deviation Score (BMI‐SDS), computed using the KiGGS age‐ and sex‐specific LMS reference for German children and adolescents (Rosario et al. 2010). The BMI‐SDS was chosen as it differentiates better between values below the first percentile. Significant correlations were defined as *p* < 0.05 after FDR correction. Sixth, for regression analyses, CTh of the precuneus (the only region showing significant group differences) was entered as the dependent variable. Predictors included age, BMI‐SDS, BAI total score, BPQ‐SF subscales (BPQ‐A, BPQ‐supra, BPQ‐sub), PAQ subscales (NDIF, PDIF, NDDF, PDDF, EOT), and selected EDI‐II subscales: From the 11 available EDI‐II subscales, we excluded Bulimia (patients were recruited without bulimic symptoms), Interoceptive Awareness (due to conceptual overlap with the BPQ), and Body Dissatisfaction, Asceticism, and Impulse Regulation (due to multicollinearity, indicated by elevated VIF values). The final EDI‐II predictors were Drive for Thinness, Perfectionism, Maturity Fears, Social Insecurity, Interpersonal Distrust, and Ineffectiveness. Multicollinearity was assessed with VIF, and predictors were selected using backward elimination (PIN = 0.05, POUT = 0.10) starting from the full theory‐driven model (Son et al. 2010).

Finally, in addition to our hypothesis‐driven analyses, exploratory analyses examined group differences in GMV, CTh, and CSA (obtained with FreeSurfer's *aparcstats2table* function). Multiple comparisons were again controlled using FDR within each metric across regions. Group differences in non‐cortical regions were assessed using *asegstats2table* and likewise FDR‐corrected. Regions that survived within‐metric FDR correction were then correlated individually with psychological assessment scores.

## 3 Results

### 3.1 Sample Characteristics

The AN group comprised 26 female adolescents aged 12–18 years (*M* = 15.04, SD = 1.46), while the control group included 24 females aged 12–19 years (*M* = 15.13, SD = 1.75). There was no statistically significant difference in age between the two groups (*p* = 0.850, *d* = −0.06). However, all other psychological characteristics differed significantly between the groups (all *p* < 0.05). A comprehensive summary of these differences is presented in Table 1. An overview of clinical and educational characteristics is presented in Table 2. Education was used as an approximation of socioeconomic status, in line with standard practice in German health monitoring studies (Rodriguez Roca et al. 2023); however, this single indicator does not capture all dimensions of socioeconomic position (e.g., income and occupational status). Race/ethnicity were not assessed, given the sensitivity of such inquiries in the German context.

**TABLE 1:** Statistical comparisons of clinical and psychological measures between AN and control groups.

Variable	AN group		Control group		Group comparisons		
	M/Mdn	SD/IQR	M/Mdn	SD/IRQ	*p* ^a^	*U/t*	Cohen's *d/r*
Age (years)	15.04	1.46	15.13	1.75	0.850	*t* = 0.19	*d* = −0.06
BMI Percentile	2.50	4.00	48.00	44.50	**< 0.001**	*U* = 10.50	*r* = −0.83
Illness duration (weeks)	45.58	50.50	—	—	—	—	
EDI‐II	292.35	71.89	191.45	47.52	**< 0.001**	*t* = −5.8	*d* = 1.64
BAI	19.00	14.50	5.00	7.75	**< 0.001**	*U* = 139.00	*r* = −0.48
BPQ‐A	64.00	48.25	49.00	40.50	**0.025**	*U* = 197.00	*r* = −0.32
BPQ‐supra	17.00	9.50	15.50	2.00	0.283	*U* = 257.50	*r* = −0.15
BPQ‐sub	10.50	6.25	7.00	3.00	**0.005**	*U* = 170.50	*r* = −0.40
BDI‐II	17.50	23.75	3.00	9.50	**< 0.001**	*U* = 70.00	*r* = −0.67
PAQ	71.00	47.50	48.50	49.50	**0.025**	*U* = 196.50	*r* = −0.32

*Note*: Significant *p*‐values are marked in bold.

Abbreviations: AN, anorexia nervosa; BAI, Beck Anxiety Inventory; BDI‐II, Beck Depression Inventory‐II; BPQ, Body Perception Questionnaire‐Short Form (awareness (BPQ‐A), supra‐diaphragmatic reactivity (BPQ‐supra), sub‐diaphragmatic reactivity (BPQ‐sub)); EDI‐II, Eating Disorder Inventory‐2; PAQ, Perth Alexithymia Questionnaire.

^a^ Comparisons were conducted using *t*‐test or Mann–Whitney U Test with a significance level of *p* < 0.05.

**TABLE 2:** Clinical and educational characteristics of AN and control groups.

Variable	AN group	Control group
	*N*	*N*
Number of participants in study	26	24
Treatment setting		
Outpatient	10	—
Inpatient	16	—
Education^a^		
Gymnasium	11	15
Realschule	6	7
Mittelschule	4	1
Fachoberschule	3	1
Berufsschule	1	—
Diagnoses^b^		
F50.0 AN restrictive	22	—
F50.1 AN atypical	4	—
F32.1 moderate depression	9	—
F33.1 moderate recurrent depression	1	—
F42.2 mixed OCD	2	—
F66.0 sexual orientation crisis	1	—
Medication and supplements		
Antidepressiant (Sertralin, Escitalopram)	3	—
Atypical Antipsychotics (Quetiapine, Olanzapine)	2	—
Vitamins and Supplements (Vitamin D, B12, Calcium)	5	—
Other medication (digestion, allergies)	5	
Birth control pill	1	3

Abbreviations: AN, anorexia nervosa; OCD, Obsessive Compulsive Disorder.

^a^ School types in Germany: Mittelschule—9 years of elementary school; Realschule—intermediate level of secondary school, regular duration of 6 years; Gymnasium—highest level of secondary school, regular duration of 8–9 years; Fachoberschule/Berufsoberschule—tertiary school to achieve advanced technical college certificate, duration of 2–3 years in addition to duration of Realschule; Berufsschule—vocational school, regular duration of 2–3 years, after secondary school.

^b^ Diagnostic classifications based on ICD‐10 codes, based on expert clinical assessments conducted in the associated clinics. Multiple diagnoses are possible.

### 3.2 Structural Group Comparisons

No significant differences (all *p* > 0.05) in volume were observed in overall brain regions, including supratentorial regions without ventricles, total gray matter, or subcortical gray matter as well as in cerebral and cerebellar white and gray matter, hippocampus, amygdala, and brainstem using permutation‐based non‐parametric testing and FDR correction. For detailed results, see Table 3.

**TABLE 3:** Global brain volume metrics in AN and control groups.

Variables	AN group		Control group		Non parametric permutation test
	Mean	SD	Mean	SD	*adj. p* ^a^
Supratentorial without Ventricles	0.71	0.08	0.76	0.10	0.243
Brainstem	0.01	< 0.01	0.01	< 0.01	0.360
Total Gray Matter	0.49	0.06	0.52	0.06	0.190
Cortex	0.36	0.04	0.39	0.05	0.190
Sub‐cortical Gray Matter	0.04	0.01	0.05	0.01	0.190
Cerebral White Matter	0.31	0.04	0.33	0.05	0.603
Cerebellum Cortex	0.08	0.01	0.09	0.01	0.375
Cerebellum White Matter	0.02	< 0.01	0.02	< 0.01	0.240
Amygdala	< 0.01	< 0.01	< 0.01	< 0.01	0.190
Hippocampus	0.01	< 0.01	0.01	< 0.01	0.375

Abbreviation: AN, anorexia nervosa.

^a^ Significance level of *p* < 0.05. *p*‐values were adjusted using FDR.

**FIGURE 1:**
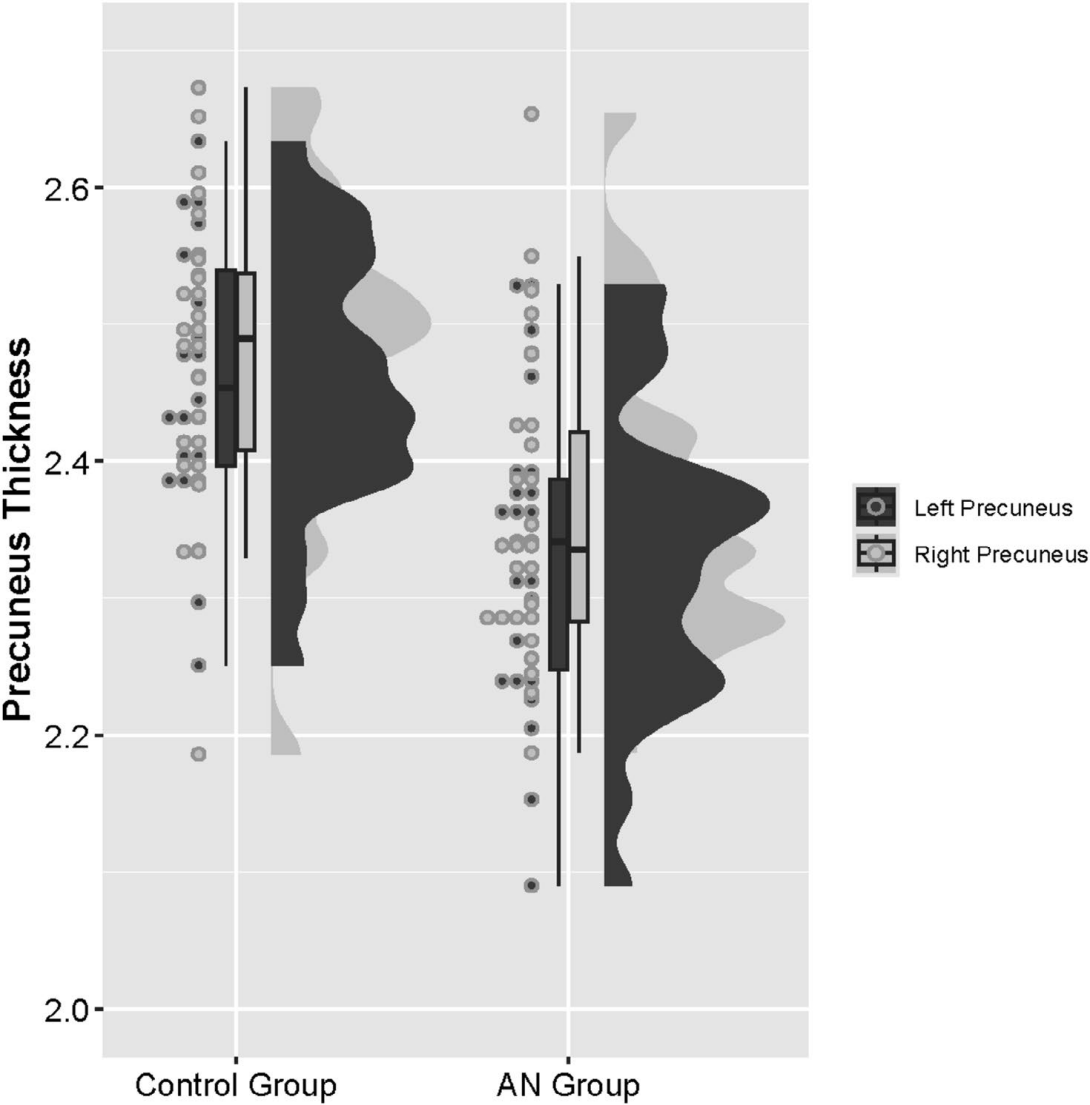
Distribution of precuneus thickness across the control and AN groups for the left and right precuneus. AN, anorexia nervosa.

**FIGURE 2:**
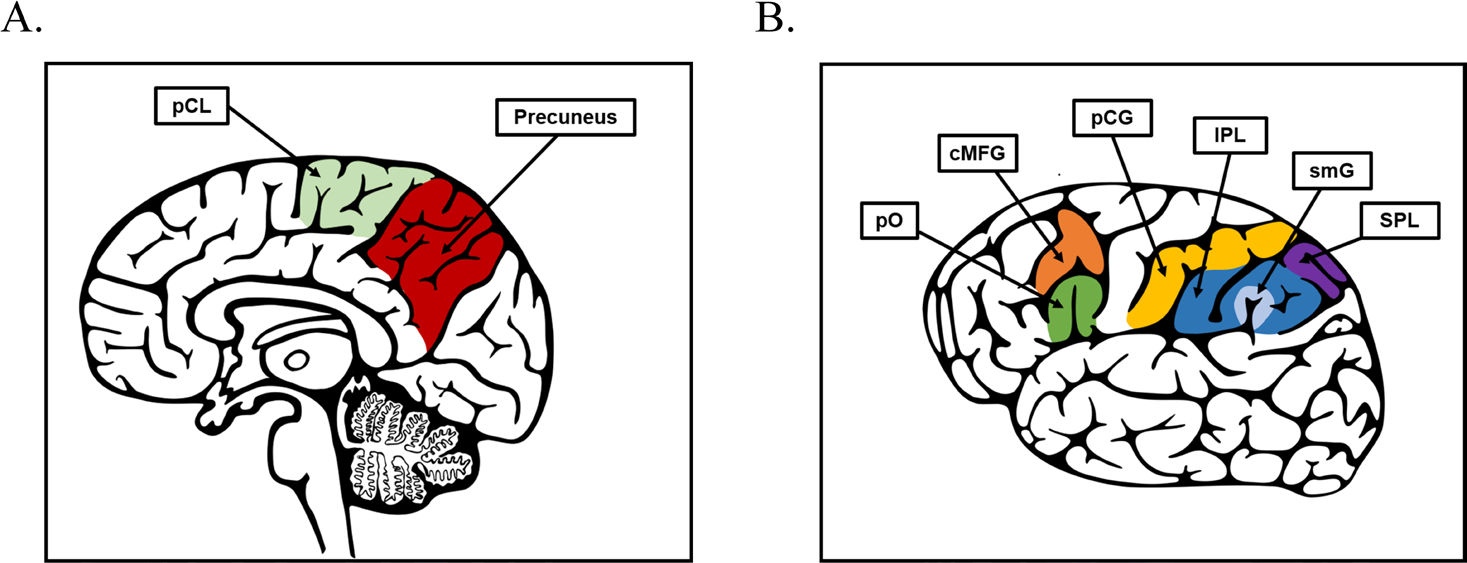
Localization of brain areas showing significant group differences in cortical thickness. (A) Sagittal view highlighting the precuneus (red), and paracentral lobule (pCL, green). (B) Lateral view highlighting the pars opercularis (pO, green), caudal middle frontal gyrus (cMFG, orange), postcentral gyrus (pCG, yellow), inferior parietal lobule (IPL, blue), supramarginal gyrus (smG, light blue), and superior parietal lobule (SPL, purple). pCL, paracentral lobule. pO, pars opercularis; cMFG, Caudal middle frontal gyrus; pCG, Postcentral gyrus; IPL, Inferior parietal lobule; smG, Supramarginal gyrus; SPL, Superior parietal lobule.

The permutation‐based non‐parametric combination of surface area and CTh revealed no significant differences between groups in the cingulate cortex or precuneus. Moreover, no significant differences were found individually examining thickness, surface area or volume in the caudal ACC, rostral ACC, PCC, or isthmus of the cingulate (IMC), except for the CTh of the precuneus (*p* = 0.003), using the permutation‐based non‐parametric approach and FDR correction. See Figure 1 for the distribution of precuneus thickness across the control and AN groups for the left and right precuneus and Figure 2A for a graphical depiction of precuneus location. For detailed results, see Table 4.

**TABLE 4:** Comparative analysis of cingulate cortex and precuneus metrics between AN and control groups.

Variable	Permutation‐based nonparametric combination		Non parametric permutation test					
			Thickness (mm)		Area (mm^a^)		Volume (mm^b^)	
	Fisher statistic^c^	*p*	*adj. p* ^a^	obs. *U* ^b^	*adj. p* ^a^	obs. *U* ^c^	*adj. p* ^a^	obs. *U* ^c^
cACC	3.01	0.549	0.814	*U* = 337	0.814	*U* = 287	0.872	*U* = 303
rACC	1.68	0.794	0.872	*U* = 302	0.814	*U* = 277	0.773	*U* = 243
PCC	3.08	0.561	0.144	*U* = 270	0.814	*U* = 270	0.814	*U* = 330
IMC	0.79	0.950	0.115	*U* = 187	0.814	*U* = 287	0.814	*U* = 264
Precuneus	2.18	0.727	**0.003**	***U* = 119**	0.814	*U* = 333	0.773	*U* = 241

*Note*: Significant results are marked in bold.

Abbreviations: cACC, caudal anterior cingulate cortex; IMC, isthmus of the cingulate; PCC, posterior cingulate cortex; rACC, rostral anterior cingulate cortex.

^a^ Adjusted *p*‐value using FDR‐correction. Significance level of *p* < 0.05.

^b^ Observed *U*‐value from the original dataset before permutation testing.

^c^ Fisher statistic of original dataset.

### 3.3 Correlation Analysis

In order to investigate the correlations between the total scores of psychological assessments (EDI‐II, BAI, BDI‐II, BPQ‐A, BPQ‐supra, BPQ‐sub, PAQ), BMI‐SDS, and the CTh of the precuneus, Kendall's tau was computed. Strong positive correlations were observed among the psychological assessments. BMI‐SDS showed moderate negative correlations with all psychological assessments (ranging from *τ* = −0.23 to *τ* = −0.37) except for the BPQ‐sub and the PAQ (*p* > 0.05). Additionally, CTh of the precuneus was moderately negatively correlated with the total scores of the psychological assessments EDI‐II, BAI, and BDI‐II (*τ* = −0.26 to *τ* = −0.37), while demonstrating a moderate positive correlation with the BMI‐SDS (*τ* = 0.33). No significant correlations were identified between precuneus CTh and the PAQ or the three BPQ scales (*p* > 0.05). For detailed results, refer to Table 5.

**TABLE 5:** Bivariate correlations between precuneus thickness and measures of psychopathology and BMI Standard Deviation Scores.

Variable		1.	2.	3.	4.	5.	6.	7.	8.	9
1. EDI‐II	Kendall's τ	1								
	*p*‐value	.								
2. BAI	Kendall's τ	0.62	1							
	*p*‐value	**< 0.001**	.							
3. BDI‐II	Kendall's τ	0.77	0.63	1						
	*p*‐value	**< 0.001**	**< 0.001**	.						
4. PAQ	Kendall's τ	0.57	0.62	0.53	1					
	*p*‐value	**< 0.001**	**< 0.001**	**< 0.001**	.					
5. BPQ‐A	Kendall's τ	0.28	0.19	0.25	0.18	1				
	*p*‐value	**0.008**	0.066	**0.019**	0.078	.				
6. BPQ‐supra	Kendall's τ	0.38	0.49	0.41	0.43	0.34	1			
	*p‐value*	**< 0.001**	**< 0.001**	**< 0.001**	**< 0.001**	**0.002**	.			
7. BPQ‐sub	Kendall's τ	0.32	0.34	0.31	0.2	0.44	0.44	1		
	*p‐value*	**0.004**	**0.003**	**0.006**	0.075	**< 0.001**	**< 0.001**	.		
8. BMI‐SDS	Kendall's τ	−0.37	−0.24	−0.34	−0.17	−0.24	−0.12	−0.31	1	
	*p*‐value	**< 0.001**	**0.021**	**0.001**	0.089	**0.028**	0.255	**0.005**	.	
9. Precuneus thickness	Kendall's τ	−0.26	−0.32	−0.25	−0.16	−0.12	−0.04	−0.19	0.33	1
	*p*‐value	**0.014**	**0.003**	**0.017**	0.124	0.255	0.702	0.079	**0.002**	.

*Note*: Correlations between precuneus, psychological assessments, and BMI Standard Deviation Scores (BMI‐SDS) are presented. Correlations remaining significant after FDR‐correction are marked in bold.

Abbreviations: BAI, Beck Anxiety Inventory; BDI‐II, Beck Depression Inventory‐II; BMI‐SDS, Body Mass Index Standard Deviation Scores; BPQ, Body Perception Questionnaire‐Short Form (awareness (BPQ‐A), supra‐diaphragmatic reactivity (BPQ‐supra), sub‐diaphragmatic reactivity (BPQ‐sub)); EDI‐II, Eating Disorder Inventory‐2; PAQ, Perth Alexithymia Questionnaire.

### 3.4 Linear Multiple Regression Model

A linear multiple regression model was conducted to predict precuneus CTh from age, BMI SDS, selected EDI‐II subscales (Drive for Thinness, Bulimia, Perfectionism, Maturity Fears, Social Insecurity, Interpersonal Distrust, Ineffectiveness), BAI total score, all three BPQ subscales, and PAQ subscales (PDIF, NDIF, PDDF, NDDF, GEOT). After backward elimination, Interpersonal Distrust (*B* = −0.02, *p* < 0.001) and BAI (*B* = −0.01, *p* < 0.001) remained significant negative predictors, while Social Insecurity (*B* = 0.01, *p* < 0.001), PAQ‐NDDF (*B* = 0.01, *p* = 0.003), and BPQ‐supra (*B* = 0.01, *p* = 0.006) were positive predictors. Drive for Thinness showed a non‐significant negative trend (*B* = −0.00, *p* = 0.055). The model explained 47% of variance (adjusted *R* ^*2*^ = 0.47) with all VIFs below 5 (see Table 6).

**TABLE 6:** Multiple regression analysis predicting precuneus thickness across both groups.

Dependent variable	Predictor	*B*	SE	*β*	*t*	*p*	VIF	CI *B*	*adj. R* ^*2*^
Precuneus thickness	Intercept	2.33	0.05	—	42.79	**< 0.001**		2.222–2.442	0.47
	EDI‐II‐DT	−0.00	0.00^a^	−0.27	−1.97	0.055	1.70	−0.005–0.000	
	EDI‐II‐ID	−0.02	0.00^a^	−0.96	−4.60	**< 0.001**	4.02	−0.023 to −0.009	
	EDI‐II‐SI	0.01	0.00^a^	0.78	3.81	**< 0.001**	3.86	0.006–0.018	
	BAI	−0.01	0.00^a^	−0.88	−4.51	**< 0.001**	3.56	−0.013 to −0.005	
	BPQ‐supra	0.01	0.00^a^	0.46	2.87	**0.006**	2.40	0.003–0.015	
	PAQ‐NDDF	0.01	0.00^a^	0.54	3.19	**0.003**	2.61	0.002–0.013	

*Note*: Initial set used all subscores of the psychological assessments as predictors. The optimal predictors were found using backward elimination. Significant results are marked in bold.

Abbreviations: *β*, Standardized coefficient; B, Unstandardized coefficient; BAI, Beck Anxiety Inventory; BPQ‐supra, BPQ supra‐diaphragmatic reactivity; CI *B*, 95% Confidence interval of *B*; EDI‐II‐DT, EDI‐II Drive for Thinness; EDI‐II‐ID, EDI‐II Interpersonal Distrust; EDI‐II‐SI, EDI‐II Social Insecurity; PAQ‐NDDF, Perth Alexithymia Questionnaire Difficulty Describing Negative Feelings; SE, Standard error; VIF, Variance Inflation Factor.

^a^ Values of 0.00 represent positive values below 0.01.

### 3.5 Sensitivity Analysis

Except for precuneus thickness, which remained significant after FDR correction with high power (power = 0.98, *p* = 0.003), all other tests of our main analysis were underpowered and should be interpreted with caution. The minimum detectable effect size was Cohen's *d* ≈ 0.79 for *n*₁ = 26 and *n*₂ = 24 at *α* = 0.05 and 80% power (Cohen 1988). For details, see Table 7.

**TABLE 7:** Group differences between AN and control group in global brain measures and cortical metrics.

Variable	*U* ^a^	adj. *p* ^b^	Power^c^
Supratentoral without Ventricles	238.00	0.243	0.28
Brainstem	253.00	0.360	0.20
Total Gray Matter	221.00	0.190	0.42
Cortex	217.00	0.190	0.45
Sub‐cortical Gray Matter	222.00	0.190	0.40
Cerebral White Matter	285.00	0.603	0.07
Cerebellum Cortex	261.00	0.375	0.14
Cerebellum White Matter	233.00	0.240	0.34
Amygdala	201.00	0.190	0.58
Hippocampus	262.00	0.375	0.14
Caudal anterior cingulate cortex area	287.00	0.814	0.08
Istmus cingulate cortex area	287.50	0.814	0.09
Posterior cingulate cortex area	343.00	0.814	0.11
Precuneus area	333.00	0.814	0.06
Rostral anterior cingulate cortex area	277.00	0.814	0.11
Caudal anterior cingulate cortex thickness	337.00	0.814	0.10
Ist muscingulate cortex thickness	187.00	0.115	0.71
Posterior cingulate cortex thickness	270.00	0.814	0.15
Precuneus thickness	119.00	**0.003**	0.98
Rostral anterior cingulate cortex thickness	302.50	0.872	0.06
Caudal anterior cingulate cortexvolume	303.00	0.872	0.07
Istmus cingulate cortex volume	264.00	0.814	0.18
Posterior cingulate cortex volume	330.00	0.814	0.06
Precuneus volume	241.00	0.773	0.30
Rostral anterior cingulate cortex volume	243.00	0.773	0.28

*Note*: Significant results are marked in bold.

^a^ Observed *U*‐value from the original dataset before permutation testing.

^b^ Significance level of *p* < 0.05. *p*‐values were adjusted using FDR after non‐parametric permutation testing.

^c^ Monte Carlo simulations were conducted with 600 simulations and 1200 permutations—values that previous studies have shown to provide a stable estimate of power targeting a significance level of 0.05 (asegstats2table 2024; Allen et al. 2008)—to estimate the power.

### 3.6 Exploratory Analyses

#### 3.6.1 Structural Group Comparisons

Exploratory analyses of global volume measures revealed no significant group differences (Table 8). In contrast, exploratory analyses of cortical regions showed significant group differences exclusively in CTh. After within‐metric FDR correction, CTh differed between groups in the caudal middle frontal gyrus (cMFG), pars opercularis (pO), postcentral gyrus (pCG), supramarginal gyrus (SMG), inferior and superior parietal lobules (IPL, SPL), and the paracentral lobule (pCL). Mean total CTh also differed between groups. No significant effects were observed for GMV or CSA following FDR correction (Table 9). See Figure 2 for a lateral and sagittal view of the brain showing regions with significant CTh differences.

**TABLE 8:** Exploratory analysis of global brain volume group differences between AN and control group.

Variable	*U* ^a^	adj. *p* ^b^
Cerebelllum White Matter volume	233	0.323
Cerebellum Cortex volume	261	0.492
Thalamus volume	235	0.328
Brain Stem volume	253	0.464
Hippocampus volume	262	0.492
Amygdala volume	201	0.135
Cortex volume	217	0.222
Cerebral White Matter volume	285	0.754
Sub‐Cortical Gray volume	222	0.222
Total Gray Volume	221	0.222
SupraTentorial Volume (No Ventricle)	238	0.34
Estimated Total Intra‐Cranial volume	329	0.754
Left Lateral Ventricle volume	408	0.222
Right Lateral Ventricle volume	459	0.068
Lateral Ventricle volume	431	0.135
Inferior lateral Ventricle volume	366	0.492
Caudate volume	289	0.754
Putamen volume	195	0.135
Pallidum volume	169	0.068
3rd Ventricle volume	429	0.135
4th Ventricle volume	352	0.611
Cerebrospinal volume	382	0.395
Accumbens area volume	257	0.492
Ventral Diencephalon volume	258	0.492
Vessel volume	372	0.222
Chlorid plexus volume	457	0.068
5th‐Ventricle volume	330	0.754
Optic‐Chiasm volume	308	0.955
Posterior Corpus Callosum volume	292	0.754
Mid Posterior Corpus Callosum volume	355	0.584
Central Corpus Callosum volume	286	0.754
Mid Anterior Corpus Callosum volume	342	0.725
Anterior Corpus Callosum volume	372	0.464

^a^ Observed *U*‐value from the original dataset before permutation testing.

^b^ Significance level of *p* < 0.05. *p*‐values were adjusted using FDR after non‐parametric permutation testing Significant *p*‐values are marked in bold.

**TABLE 9:** Exploratory analysis of cortical brain metrics group differences between AN and control group.

Region	Area		Thickness		Volume	
	*U* ^a^	adj. *p* ^b^	U^a^	adj. *p* ^b^	U^a^	adj. *p* ^b^
Banks of the Superior Temporal Sulcus	291	0.906	202	0.075	262	0.620
Caudal Anterior Cingulate Cortex	287	0.906	337	0.753	303	0.923
Caudal Middle Frontal Gyrus	312	0.985	173	**0.029**	269	0.672
Cuneus	211.5	0.452	283	0.753	240	0.433
Entorhinal Cortex	316.5	0.930	368	0.522	346	0.749
Fusiform Gyrus	246	0.745	280.5	0.723	284	0.807
Inferior Parietal Lobule	325	0.906	137.5	**0.010**	252	0.514
Inferior Temporal Gyrus	235	0.635	265.5	0.643	235	0.427
Isthmus Cingulate Cortex	287.5	0.906	187	0.052	264	0.620
Lateral Occipital Cortex	191	0.451	274	0.698	179	0.156
Lateral Orbitofrontal Cortex	324	0.906	307.5	0.923	311	0.991
Lingual Gyrus	225	0.614	337	0.753	289	0.826
Medial Orbitofrontal Cortex	324	0.906	313	0.923	312	0.991
Middle Temporal Gyrus	263	0.906	250.5	0.446	250	0.511
Parahippocampal Gyrus	291	0.906	320	0.923	329.5	0.826
Paracentral Lobule	294	0.906	167.5	**0.03**	215	0.333
Pars Opercularis (Inferior Frontal Gyrus)	208	0.452	171.5	**0.03**	172.5	0.156
Pars Triangularis (Inferior Frontal Gyrus)	232.5	0.635	239.5	0.354	231	0.414
Pars Orbitalis (Inferior Frontal Gyrus)	257.5	0.837	235	0.315	182	0.156
Pericalcarine Cortex	209	0.452	355	0.658	275	0.749
Postcentral Gyrus	289	0.906	150	**0.016**	208	0.247
Posterior Cingulate Cortex	343	0.906	270	0.672	330	0.826
Precentral Gyrus	317	0.930	245	0.421	282	0.807
Precuneus	333	0.906	119	**0.007**	241	0.432
Rostral Anterior Cingulate Cortex	277	0.906	302.5	0.923	243	0.467
Rostral Middle Frontal Gyrus	248	0.745	197	0.068	200	0.220
Superior Frontal Gyrus	266	0.906	189	0.052	195	0.220
Superior Parietal Lobule	299	0.906	157	**0.021**	238	0.426
Superior Temporal Gyrus	288	0.906	188.5	0.054	230	0.414
Supramarginal Gyrus	325.5	0.906	157.5	**0.027**	286	0.815
Frontal Pole	248	0.745	291	0.755	222	0.349
Temporal Pole	279.5	0.906	349	0.698	296	0.850
Transverse Temporal Gyrus	228	0.614	345.5	0.723	291.5	0.826
Insula (Insular Cortex)	278.5	0.906	339	0.753	262	0.620
Mean Total Cortical	—	—	181	**0.047**	—	—

*Note*: Significant *p*‐values are marked in bold.

^a^ Observed *U*‐value from the original dataset before permutation testing.

^b^ Significance level of *p* < 0.05. *p*‐values were adjusted using FDR after non‐parametric permutation testing.

#### 3.6.2 Correlation Analysis

After FDR correction, significant negative correlations emerged between the EDI‐2 total score and both the IPL and precuneus. The BAI total score showed negative correlations with the cMFG, IPL, pCL, pO, precuneus, SPL, and mean total CTh. Similarly, the BDI total score correlated negatively with the IPL, pCL, pO, pCG, precuneus, and SMG. The BPQ‐sub score was negatively associated with the cMFG and SMG. In contrast, positive correlations with BMI‐SDS were observed for the cMFG, pO, pCG, SMG, IPL, SPL, pCL, and mean total CTh (Table 10).

**TABLE 10:** Exploratory analysis of correlations between cortical thickness in brain regions with FDR‐significant group differences and measures of psychopathology and BMI Standard Deviation Scores.

Brain region thickness	Metric	EDI‐II	BAI	BDI‐II	PAQ	BPQ‐ A	BPQ‐supra	BPQ‐sub	BMI‐SDS
cMFG	Kendall's τ	−0.20	−0.24	−0.18	−0.11	−0.20	−0.09	−0.33	0.30
	*p*‐value	0.088	**0.046**	0.121	0.312	0.088	0.417	**0.022**	**0.023**
IPL	Kendall's τ	−0.24	−0.29	−0.28	−0.15	−0.14	−0.04	−0.21	0.34
	*p*‐value	**0.046**	**0.027**	**0.028**	0.202	0.226	0.739	0.088	**0.022**
pCL	Kendall's τ	−0.22	−0.24	−0.19	−0.05	−0.22	−0.01	−0.22	0.29
	*p*‐value	0.288	**0.034**	0.222	0.448	0.245	0.417	0.245	**0.026**
pO	Kendall's τ	−0.12	−0.27	−0.14	−0.08	−0.13	−0.1	−0.14	0.26
	*p*‐value	0.068	**0.048**	0.109	0.715	0.064	0.953	0.088	**0.034**
pCG	Kendall's τ	−0.13	−0.19	−0.16	−0.03	−0.15	0.02	−0.23	0.30
	*p*‐value	0.252	0.109	0.182	0.795	0.182	0.837	0.064	**0.022**
Precuneus	Kendall's τ	−0.26	−0.32	−0.25	−0.16	−0.12	−0.04	−0.19	0.33
	*p*‐value	**0.036**	**0.022**	**0.039**	0.182	0.273	0.755	0.121	**0.022**
SPL	Kendall's τ	−0.20	−0.29	−0.24	−0.12	−0.14	−0.02	−0.10	0.26
	*p*‐value	0.08	**0.026**	0.053	0.273	0.232	0.837	0.379	**0.035**
smG	Kendall's τ	−0.23	−0.23	−0.26	−0.11	−0.14	−0.09	−0.29	0.31
	*p*‐value	0.057	0.057	**0.040**	0.347	0.221	0.448	**0.030**	**0.022**
Mean total Cortical	Kendall's τ	−0.20	−0.28	−0.19	−0.16	−0.17	−0.03	−0.21	0.27
	*p*‐value	0.088	**0.030**	0.109	0.163	0.159	0.820	0.090	**0.030**

*Note*: Correlations between brain regions with FDR‐significant group differences, psychological assessments, and Body Mass Index Standard Deviation Scores (BMI‐SDS) are presented. Correlations remaining significant after FDR‐correction are marked in bold.

Abbreviations: BAI, Beck Anxiety Inventory; BDI‐II, Beck Depression Inventory‐II; BPQ, Body Perception Questionnaire‐Short Form: awareness (BPQ‐A), supra‐diaphragmatic reactivity (BPQ‐supra), sub‐diaphragmatic reactivity (BPQ‐sub); cMFG, caudal middle frontal gyrus; EDI‐II, Eating Disorder Inventory‐2; IPL, inferior parietal lobules; PAQ, Perth Alexithymia Questionnaire; pCG, postcentral gyrus; pCL, paracentral lobule; pO, pars opercularis; smG, supramarginal gyrus; SPL, superior parietal lobules.

#### 3.6.3 Sensitivity Analysis

All significant CTh differences during our exploratory analysis were sufficiently powered (power > 0.79). For details, see Table 11.

**TABLE 11:** Sensitivity analysis of significantly different brain regions during exploratory analysis.

Brain region thickness	*U* ^a^	adj. *p* ^b^	Power^c^
Caudal Middle Frontal Gyrus	173	0.029	0.82
Inferior Parietal Lobule	137.5	0.010	0.95
Paracentral Lobule	167.5	0.030	0.85
Pars Opercularis (Inferior Frontal Gyrus)	171.5	0.030	0.81
Postcentral Gyrus	150	0.016	0.91
Superior Parietal Lobule	157	0.021	0.88
Supramarginal Gyrus	157.5	0.027	0.89
Mean Total Cortical	181	0.047	0.81

*Note*: Significant results are marked in bold.

^a^ Observed *U*‐value from the original dataset before permutation testing.

^b^ Significance level of *p* < 0.05. *p*‐values were adjusted using FDR after non‐parametric permutation testing.

^c^ Monte Carlo simulations were conducted with 600 simulations and 1200 permutations—values that previous studies have shown to provide a stable estimate of power targeting a significance level of 0.05 (asegstats2table 2024; Allen et al. 2008)—to estimate the power.

## 4 Discussion

This study investigated structural brain alterations in adolescent females with AN, focusing specifically on regions within the cingulate cortex and precuneus and their association with the psychopathology of AN. We hypothesized that individuals with AN would exhibit reduced GMV, CTh, and CSA in the above‐mentioned regions compared to controls and that these alterations would correlate with psychological assessment scores.

Our findings partially supported these hypotheses, revealing a significant reduction in CTh of the precuneus in the AN group compared to the control group, with significant associations to psychological assessment subscores. However, no significant structural differences were observed in other cingulate cortex regions or global brain parameters. Exploratory analyses showed additional CTh reductions in selected parietal and frontal regions.

The observed reduction in precuneus CTh aligns with prior research highlighting its role in self‐referential processing, body image perception, and aspects of consciousness, domains frequently disrupted in AN (Zhang et al. 2018; Cavanna and Trimble 2006). As a key hub within the DMN, the precuneus is involved in self‐reflection and self‐monitoring.

In contrast to previous studies, we did not find significant structural differences in the ACC, MCC, PCC, or IMC between the AN group and the control group. Prior research has reported reductions in GMV in these regions among individuals with AN (Yu et al. 2024; Walton et al. 2022; Zhang et al. 2018; Su et al. 2021; Joos et al. 2010; Gaudio et al. 2011). Additionally, no significant group differences were found in global brain measures, including cortical and cerebral white matter, subcortical structures, and the cerebellum (Myrvang et al. 2020; Katzman et al. 1996). These discrepancies may be attributed to variations in sample characteristics, such as age and illness duration, or methodological differences. Our sample comprised adolescents with a relatively short illness duration, which may result in less pronounced structural changes compared to adults with chronic AN. Furthermore, our use of the non‐parametric combination (NPC) method, which integrates CTh and CSA metrics, may have increased the specificity of our findings by reducing susceptibility to artifacts commonly encountered in traditional volumetric analyses (Winkler et al. 2016).

Our correlation analyses revealed that the CTh of the precuneus was moderately negatively correlated with psychological assessments of eating disorder symptomatology (EDI‐II), anxiety (BAI), and depression (BDI‐II), and positively correlated with BMI‐SDS. These findings suggest that greater scores in these psychological tests are associated with reduced precuneus CTh, which could reflect the neurobiological underpinnings of the clinical symptoms observed in AN. The positive correlation with the BMI‐SDS could indicate that nutritional status influences brain structure, consistent with the effects of malnutrition on brain development and function (Kaye et al. 2009). Moreover, our multiple regression analysis provided further exploratory insights into the relationship between test scores and precuneus CTh. Higher scores on the “Interpersonal Distrust” subscale of the EDI‐II—a reflection of a sense of alienation and reluctance to form close relationships (Garner et al. 1983)—were significantly negatively associated with precuneus' CTh. This suggests that difficulties in trusting others could be linked to greater reductions in CTh in the precuneus. This aligns with the role of the precuneus in self‐perception and social cognition.

In contrast, the EDI‐II “Social Insecurity” subscale, characterized by heightened insecurity in social situations and fear of negative evaluation (Garner et al. 1983), the PAQ subscale “Difficulties Describing Negative Feelings”, which reflects difficulty in expressing negative emotions, and the BPQ supra‐diaphragmatic reactivity subscale each showed positive correlations with precuneus CTh. These findings are somewhat counterintuitive, as reduced CTh in the precuneus has been linked to greater symptom severity in our sample. This pattern may suggest opposing effects of psychological dimensions on brain structure, although directionality cannot be inferred and could also be reversed. The observed positive associations could reflect compensatory neuroplasticity processes or developmental adaptations in response to specific psychopathological traits. Moreover, increased CTh within a frontoparietal network has been reported in individuals with social anxiety disorder, which may explain the link between greater “Social Insecurity” (EDI‐II) scores and precuneus' CTh (Brühl et al. 2014).

Furthermore, previous research done by Demers and colleagues found a positive correlation between CTh of the dorsal anterior cingulate cortex (dACC) and alexithymia scores in patients with childhood trauma‐related PTSD (Demers et al. 2015). This association has been attributed to impaired emotional processing, potentially leading to reduced synaptic pruning and increased CTh (Demers et al. 2015). Paralleling these findings, elevated BPQ supra‐diaphragmatic reactivity—which captures autonomic symptoms above the diaphragm (e.g., chest tightness, palpitations, shortness of breath) that may serve as unrecognized or misinterpreted signals of emotional arousal—could likewise reflect disrupted interoceptive awareness contributing to altered cortical maturation in AN. If the dACC undergoes experience‐dependent synaptic pruning.

Exploratory analyses outside our primary hypothesis revealed CTh alterations in frontal (cMFG, pO) and parietal regions (pCG, SMG, IPL, SPL, pCL) as well as reduced mean total CTh. These findings largely replicate previous large‐scale studies in young adults (Walton et al. 2022) and children (Moreau et al. 2025). Functionally, frontal regions such as the cMFG and pO are central to set‐shifting and inhibitory control (Zastrow et al. 2009; Petrides and Pandya 1999; Moll et al. 2002; Lao‐Kaim et al. 2015; Aron 2011; Brooks et al. 2011), while parietal regions support body‐related visual and somatosensory processing (Felician et al. 2004; Case et al. 2012; Gaudio and Quattrocchi 2012; Vocks et al. 2010; Wagner et al. 2013; Favaro et al. 2014; Castellini et al. 2013). Together, these alterations may contribute to both impaired behavioral control and distorted body perception in AN.

Correlational analyses further supported this interpretation. Higher eating disorder symptoms (EDI‐II) were associated with reduced CTh in the IPL and precuneus, consistent with previous work (Westwater et al. 2018; Lee et al. 2014). Anxiety and depression scores showed widespread negative associations with frontal and parietal regions, in line with prior structural studies (Miskovich et al. 2016; Kim et al. 2022; Nan et al. 2022; Bitsika et al. 2023; Forster et al. 2015; Liang et al. 2022; Hamilton et al. 2025; Schultz et al. 2019; Hwang et al. 2015). Notably, mean total CTh correlated only with the BAI, a finding we interpret cautiously, as many BAI items reflect somatic complications of AN (Friars et al. 2023; Jafar and Morgan 2021; Franques et al. 2017; Mathew and Thoppil 2025; Jenkins et al. 2021), rather than anxiety per se. Finally, BPQ‐sub scores correlated with cMFG and SMG, consistent with the role of parietal regions in interoceptive awareness and bodily integration (Lavagnino et al. 2014).

BMI‐SDS was positively associated with CTh across multiple regions, including the cMFG, IPL, SPL, SMG, and mean total CTh, reinforcing the strong influence of nutritional status on cortical morphology (Kaye et al. 2009). These exploratory findings, while secondary to our primary analyses, suggest broader frontal and parietal alterations mapping onto control and body perception, and highlight the importance of nutritional rehabilitation in adolescent AN.

Several limitations of our study should be acknowledged. The small sample size may limit statistical power and generalizability, though recruiting adolescent participants, particularly in clinical populations like AN, is challenging. Furthermore, most analyses were underpowered (most power < 0.70) and should be considered with caution; however, the precuneus thickness effect remained significant after FDR and had high power (power = 0.98), supporting the effect's robustness. Our focus on recently ill patients adds further constraints, yet the sample remains notable and provides valuable insights. The cross‐sectional design prevents determining whether brain structure alterations precede or result from psychopathology. Additionally, we did not control for factors like physical activity or medication use, though most reported medications were supplements, and too few participants took psychotropic medications for meaningful statistical control. Furthermore, given the overfitting risk and possible instability of backward elimination, our model should be interpreted as exploratory rather than confirmatory (Field 2018).

Despite these limitations, our study has key strengths. We reduced variability by including only female adolescents and focusing on a specific brain region. Advanced techniques, including high‐resolution 3 Tesla Siemens MRI with a 64‐channel head coil and permutation‐based NPC, ensured precise, reliable brain metric measurements. Our multidisciplinary approach, integrating structural imaging and psychological assessment, enhances understanding of neurobiological and psychological dimensions in AN. A well‐matched control group strengthens the study's validity, allowing accurate comparisons.

Future research should incorporate functional neuroimaging to explore the functional significance of structural alterations. Examining comorbid psychiatric conditions and their impact on brain structure would deepen our understanding of neurobiology–psychopathology interactions. Additionally, including recovered individuals could clarify whether neurobiological changes are reversible and distinguish between state‐ and trait‐related alterations in AN.

## 5 Conclusion

This study provides new insights into the neurobiological underpinnings of AN, demonstrating significant reductions in precuneus CTh and its associations with key psychological dimensions. These findings highlight the role of the precuneus in self‐referential processing, body image perception, and emotional regulation. While reduced thickness correlated with greater eating disorder symptomatology, and anxiety, unexpected positive associations with social insecurity, autonomic nervous system reactivity above the diaphragm, and alexithymia suggest a complex interplay between starvation‐related cortical thinning and potential delays in synaptic pruning.

Exploratory analyses further indicated broader alterations, including reduced mean CTh and changes in frontal and parietal regions, while global volumetric measures remained unaffected. Together, these results emphasize the importance of both focal and widespread cortical changes in AN. Despite limitations of sample size and cross‐sectional design, the use of advanced neuroimaging and psychological integration strengthens our findings, and future longitudinal studies are needed to clarify their functional and clinical relevance for treatment and recovery.

## Author Contributions

**Irina Jarvers:** conceptualization, methodology, software, validation, formal analysis, data curation, project administration, writing – original draft, writing – review and editing, visualization, funding acquisition. **Raphael Degmayr:** methodology, software, validation, formal analysis, data curation, writing – original draft. **Alexandra Otto:** conceptualization, investigation, validation, writing – review and editing. **Ricarda Jacob:** conceptualization, investigation, validation, writing – review and editing. **Wilhelm Malloni:** conceptualization, data curation, validation, writing – review and editing. **Stephanie Kandsperger:** conceptualization, investigation, validation, writing – review and editing. **Daniel Schleicher:** conceptualization, validation, writing – review and editing. **Angelika Ecker:** conceptualization, validation, writing – review and editing. **Isabel Wiesinger:** conceptualization, validation, writing – review and editing. **Christina Wendl:** conceptualization, validation, writing – review and editing. **Mark Greenlee:** conceptualization, resources, writing – review and editing, supervision. **Romuald Brunner:** conceptualization, resources, writing – review and editing, supervision, project administration.

## Ethics Statement

All procedures involving human participants were approved by the Ethics Committee of the University of Regensburg on the 23rd of June, 2021 (reference number: 21‐2438‐101).

## Conflicts of Interest

The authors declare no conflicts of interest.

## Data Availability

The data that support the findings of this study are available from the corresponding author, Irina Jarvers, upon reasonable request.
